# Supplementary material for: Sphenofontis velserae gen. et sp. nov., a new rhynchocephalian from the Late Jurassic of Brunn (Solnhofen Archipelago, southern Germany)
Source: PeerJ. 2021 May 3;9:e11363. doi: 10.7717/peerj.11363 (PMC8101455; doi:10.7717/peerj.11363)
Supplement: Supplemental Information 1 [file peerj-09-11363-s001.docx]

***Sphenofontis velserae* gen. et sp. nov., a new rhynchocephalian from the Late Jurassic of Brunn (Solnhofen Archipelago, southern Germany)**

**Supplemental Data S1**

A preliminary phylogenetic analysis was conducted to test the taxonomic position of *Sphenofontis velserae* gen. et sp. nov. We based the analysis on the reduced (35 operational taxonomic units) matrix published by Simões et al. (2020), adding the new taxon in Mesquite v3.61 (Maddison & Maddison, 2017) with the following scorings:

Sphenofontis 0101?????10??0?011??1??0?1??????00??0???11??22?1011010110?111010011?0000-111021-?00111-12100?10?00210?100311?010?1?11??01000?10?111

Two maximum parsimony analyses were run in TnT v1.5 (Goloboff et al., 2008) with the same parameters used by Simões et al. (2020), the first one using equal weigths and the second one using implied weigthing with a concavity index (K) = 12. Results were largely comparable between the two analyses and between them and the results of the same analyses presented by Simões et al. (2020). The only differences are in the position of *Kallimodon pulchellus* and in the resolution of a few taxa. However, our scope here was focused only on the new taxon, and so we will not discuss on these differences in depth. *Sphenofontis* is always recovered as sister to a clade including *Cynosphenodon*, *Kawasphenodon*, and *Sphenodon* (i.e., Sphenodontinae sensu Simões et al., 2020), thus supporting our taxonomic conlcusions.

Specific data for the two analyses are as follows:

1. The first run using equal weigths resulted in 32 equally-parsimonious trees. The strict consensus trees was 290 steps long. The consistency index (CI) is 0.500 and the retention index (RI) is 0.651
2. The analysis with implied weigthing resulted in three equally-parsimonious trees, the strict consensus of which is 286 steps long. For this, CI = 0.507, and RI = 0.661

**References**

Goloboff PA, Farris JS, Nixon KC. 2008. TNT, a free program for phylogenetic analysis. *Cladistics* 24:774-786.

Maddison W P, Maddison DR. 2017. Mesquite: a modular system for evolutionary analysis. Available at http://mesquiteproject.org

Simões TR, Caldwell MW, Pierce SE. 2020. Sphenodontian phylogeny and the impact of model choice in Bayesian morphological clock estimates of divergence times and evolutionary rates. *BMC Biology* 18:191.
